# Uncommon manifestations of *Listeria monocytogenes* infection

**DOI:** 10.1186/s12879-014-0641-x

**Published:** 2014-12-03

**Authors:** Ruchir Chavada, Caitlin Keighley, Syed Quadri, Ray Asghari, Ann Hofmeyr, Hong Foo

**Affiliations:** Department of Microbiology and Infectious Diseases, Sydney South Western Area Pathology Services (SSWPS), Liverpool Hospital, Corner Goulburn Street, Liverpool, 2170 NSW Australia; Department of Medical Oncology, Bankstown Hospital, Bankstown, 2200 NSW Australia

**Keywords:** Listeria monocytogenes, Prosthetic joint infection, Prosthetic vascular graft infection, Perianal abscess, Listeria typing, Sporadic infection

## Abstract

**Background:**

*Listeria monocytogenes* causes gastroenteritis, meningitis and bacteraemia in immunocompromised, pregnant patients, the elderly as well in immunocompetent patients. Focal infections with this organism are uncommon, especially in sporadic (non-outbreak) setting, require high index of suspicion and are challenging to diagnose. We present 3 cases of *Listeria monocytogenes* presenting as focal infections to our hospitals, all of which are the first reported cases from Australia.

**Case presentation:**

Three unrelated cases of unique focal infections caused by Listeria monocytogenes are presented. 1) A 73 year old Caucasian lady on immunosuppression for colorectal cancer presented with prosthetic knee joint septic arthritis, 2) An 83 year old Caucasian man presented with prosthetic vascular graft infection and 3) A 60 year old Asian man with perianal abscess. Except for case 1, the other cases had a prolonged duration of symptoms on presentation. Listeria was not thought to be causative organism in any of these cases until microbiological specimens isolated the organism. Matrix Associated Laser Desorption/Ionization-Time of Flight Mass Spectrometry (MALDI-TOF MS) assisted in making an earlier diagnosis of the infection in all three cases. All of these patients had Listeria monocytogenes isolated from clinical specimens. They were managed with antibiotics and surgery with favourable outcomes. Public health investigations to determine any dietary association were done, however no intervention was thought to be necessary in any of the cases except provide dietary advice. The first two cases highlight the importance of microbiological sampling in serious infections for definitive antibiotic therapy to be administered.

**Conclusion:**

Sporadic focal infections with *Listeria* occur infrequently and are often not diagnosed till culture results from microbiological specimens become available. Dietary history should be an important aspect of thorough clinical history and food consumption advice is crucial in immunocompromised patients on similar lines as given to pregnant women about listeriosis.

**Electronic supplementary material:**

The online version of this article (doi:10.1186/s12879-014-0641-x) contains supplementary material, which is available to authorized users.

## Background

Listeriosis is a serious food borne disease caused by the bacterium *Listeria monocytogenes*, affecting mostly pregnant women, neonates, the elderly and immune-compromised hosts with relatively high mortality rates in these groups. Invasive infections with Listeria may cause life threatening meningitis and bacteraemia in these groups. However it may also manifest as self-limiting gastrointestinal illness in immunocompetent hosts. A multitude of focal infections with *Listeria monocytogenes* have been reported in the literature affecting various organs [1]. There have been multi-jurisdictional and statewide outbreaks of listeriosis in Australia associated with contaminated food items in the past. However sporadic cases occur more often than cluster outbreaks [2],[3]. The source of infection in sporadic cases remains largely unknown. We present three epidemiologically unrelated cases of sporadic origin presenting as focal infections.

## Case presentation

### Case 1

**Figure 1:**
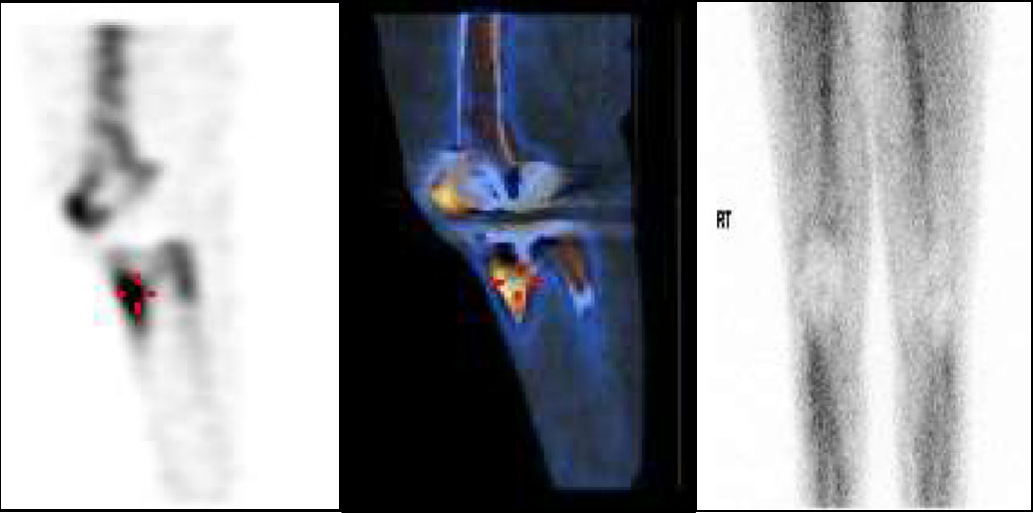
**Technetium-99 m bone scan depicting increased uptake in the region of tibial tuberosity of the prosthetic knee joint (Images 1 and 2-red pointer denotes increased uptake in the region indicative of synovitis).** Image 3 is suggestive of hyperaemia in the right knee which is also indicative of the same.

A 73 year old Caucasian woman presented with acute onset of right knee pain for 1 week. She had a background of metastatic colorectal carcinoma for which she was receiving 5-fluorouracil and oxiplatin chemotherapy, bilateral total knee replacements and type 2 diabetes. There was a preceding history of gastrointestinal illness 3 weeks prior to current presentation. A technetium 99 m-bone scan (Figure 1) demonstrated increased tracer uptake in the right knee suggestive of a septic arthritis. On arthroscopic washout of the knee, microscopy of the aspirated fluid revealed >109200 × 10^6^/L polymorphs and gram-positive rods. Cultures of the aspirate confirmed *Listeria monocytogenes* on horse blood agar at 3 days. The minimum inhibitory concentration (MIC) of penicillin tested by Etest® (AB Biodisk; Solna, Sweden) was 0.5 mg/L. Blood cultures were negative. The patient completed a 2 week course of intravenous ampicillin (2 grams every 4 hours) followed by benzyl penicillin (14.4 gram/daily) as intravenous infusion and then completed a 6 month course of [oral] amoxicillin (1 gram/TDS). She remained asymptomatic at the end of therapy.

The local public health unit was notified. On telephonic interview with the patient they found that she had a penchant for raw meat; an environmental investigation was not undertaken as this was deemed to be a sporadic case.

### Case 2

**Figure 2:**
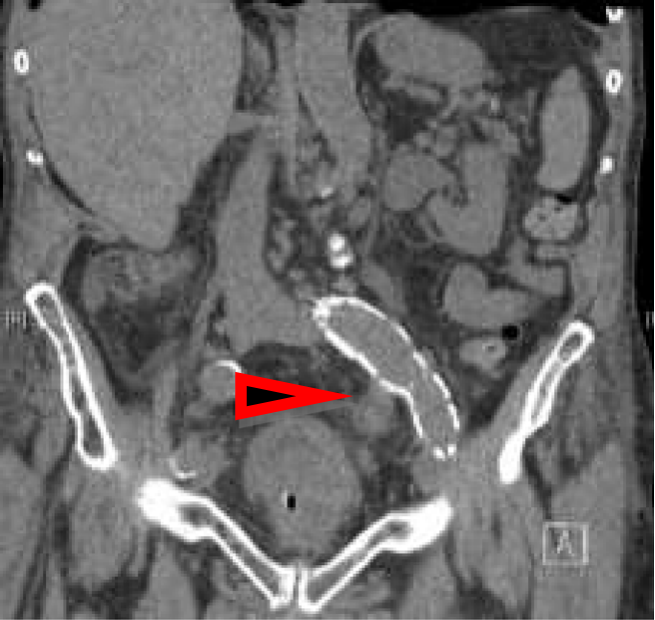
**Abdominal CT scan showing the left femoro-popliteal graft and surrounding collection.** (See arrow).

An 86 year old Caucasian man with history of peripheral vascular disease and a prosthetic (polytetra-fluroeythelene) left femoro-popliteal bypass graft in 2011, atrial fibrillation and mild mitral regurgitation presented with a discharging skin sinus in the left groin. He also had abdominal pain, fever and a raised neutrophil count (14600 × 10^6^/L) on admission. An abdominal computerized tomographic (CT)) revealed left femoro-popliteal graft leak with haematoma formation which was in close proximity to the graft (Figure 2). The patient subsequently underwent an operative excision of the femoro-popliteal graft with ovine graft replacement and femoral endartectomy. *Proteus mirabilis* and *Listeria monocytogenes* were isolated from the operative samples of peri-prosthetic fluid and graft tissue. He was commenced on intravenous ampicillin (2 grams every 4 hours) to which both of the above organisms tested sensitive [MIC of ampicillin against *Proteus mirabilis* <2 mg/L by Vitek2, (bioMerieux, Marcy-l’Etoile, France) and MIC of penicillin against *Listeria monocytogenes* was 0.5 mg/L by Etest]. His post-operative course was complicated by a surgical site infection with an extended spectrum β lactamase (ESBL) producing *Enterobacter cloacae*. He required a subsequent washout and addition of meropenem (1 gram/TDS) to the antibiotic regimen. He had a good clinical response after 6 weeks of parenteral antibiotic therapy. In follow-up, he was clinically stable and a management plan was made in consultation with patient’s surgeon and patient himself for long term oral amoxicillin (500 mg/TDS) in view of the partial retention of the infected prosthetic graft.

This patient, although elderly was not immunosuppressed with other medical conditions and no clear risk factors were identified for *Listeria* infection apart from consuming meats from a local delicatessen. He was too deemed as a sporadic case by the public health unit.

### Case 3

A 60 year old Asian man was admitted with a perianal abscess present for 3 weeks prior to presentation. He had a history of atrial fibrillation and was on oral flecainide. There were no concurrent neurological symptoms or any history of underlying immunosuppression. He underwent a surgical incision and drainage of the perianal abscess which revealed positive microbiological culture of *Listeria monocytogenes* and mixed bowel flora. The MIC of penicillin against this organism tested by Etest was 0.5 mg/L. A brain CT did not demonstrate the presence of any cerebral abscesses. The patient received a 10 day course of oral ampicillin (1 gram/TDS) and ciprofloxacin (500 mg/BD).

The public health unit was notified, however apart from an interview with the patient, no further investigation was carried out as the case did not meet the case definition of listeriosis.

## Discussion

We performed PubMed search with terms “*Listeria monocytogenes*”, “prosthetic joint septic arthritis”, “prosthetic vascular graft infection” and “perianal abscess” and reviewed articles in literature. To the best of our knowledge, these are the first reported cases of prosthetic joint septic arthritis, prosthetic vascular graft infection and perianal abscess due to *Listeria monocytogenes* in Australia. None of these 3 cases appear to be epidemiologically linked to each other nor were a part of an outbreak that occurred in Australia in 2011–2012 [3],[4].

*Listeria monocytogenes* is a gram-positive rod that is ubiquitous in the environment; it is present on unwashed vegetables and also found from foods that have been contaminated after processing. Listeriosis has a mortality rate of 10-44% in food borne outbreaks. In Australia the incidence of listeriosis is 2.5-3.6 cases per million of population per annum [5]. The Australian national case notifiable definition of ‘listeriosis’ requires laboratory evidence in the form of isolation or detection of *Listeria monocytogenes* from a site that is normally sterile, including meconium [6]. Clinical and epidemiological evidence is not required. Although similar to the Centre of Disease Control and Prevention (CDC, Atlanta) case definition, this differs from the European Centre of Communicable disease control (ECDC) definition which takes into account all three- clinical, laboratory and epidemiological criteria and subsequently classifies cases into probable and confirmed [7],[8]. CDC defines ‘invasive infection’ when the bacteria has spread beyond the gastrointestinal tract [7]. Invasive infections in pregnant women can manifest as premature delivery, miscarriages and stillbirth and as meningitis, cerebral abscess and/or septicaemia in immunocompromised patients. Patients with underlying malignancy, CNS involvement and ≥ 80 years have increased risk of mortality from invasive listeriosis [9].

According to the New South Wales (NSW) Health Department statistics of the 21 cases of listeriosis in 2011, 61% of patients were over 65 years and had underlying immunosuppression compared to 24% patients who were 65 and under [10]. A similar incidence of invasive listeriosis has been found in the United States with 24% of patients under 65 on some form of immunosuppressive therapy (glucocorticoids or chemo-radio therapy) [11]. Solid organ or haematological malignancy is also an established risk factor for listeriosis, with certain conditions like chronic lymphocytic leukaemia being associated with >1000 fold increased risk compared to the general population [9],[12]. In NSW during 2011–2012, it was found that in patients who developed invasive listeriosis 24% had history of malignancy and 28% were on immunosuppressant medications [Musto J, Health Protection NSW, personal communication].

Septic arthritis is an unusual manifestation of infection with *L. monocytogenes* with less than 80 reported cases in the literature. In the largest series of 43 patients from France, the most frequently involved joint involved was the hip (60%) followed by the knee (21%) [13]. In these patients 84% had associated orthopaedic devices, and 26% had underlying malignancy, as in our first case. Subacute infection with a median duration of 4 weeks occurred in the majority. There were no fatal cases. Other case reports with septic arthritis have demonstrated a link with tumour necrosis factor (TNF) inhibitors, which was not present in case 1 [14].

Prosthetic graft infections caused by *Listeria species* are rare, with only 8 cases reported in world literature, of which 6 patients were elderly or had underlying immunosuppression [15]. In all these cases haematogenous dissemination was the mode of infection, as was thought to be the case in our second patient, and the organism was less frequently isolated in blood culture than fluid or tissue from the graft site. Most patients were treated with definitive surgery as was case 2.

*Listeria* can cause abscess formation, the most common sites being brain and liver. Only two cases of perianal abscesses have been described so far in literature from a Danish series [16]. Both these patients died as a result of septicaemia. Our case 3 was quite well after surgical drainage of the abscess with a short course of oral antibiotics.

Laboratory confirmation of listeriosis requires isolation or detection of *L. monocytogenes* from a site that is normally sterile. A presumptive identification of *Listeria* can be made on basis of typical short Gram positive rod with characteristic “tumbling motility” by light microscopy (at room temperature but not at 37°C) and aesculin hydrolysis. The availability of Matrix-assisted Laser Desorption/Ionization Time-of-Flight Mass Spectrometry (MALDI-TOF MS) has made confirmation of *Listeria monocytogenes* relatively quick in laboratories using it. In our laboratory MALDI-TOF MS has been validated for this purpose [17]. In addition to mass spectrometry we also confirm the identification of the organism by phenotypic strip test (API Coryne®, bioMerieux, Marcy l’Etoile, France). Various methods of antibiotic susceptibility testing like Etest strips and disk diffusion have been used. We routinely employ an Etest strip on Mueller Hinton agar with laked horse blood for penicillin (0.016-256 mg/L) and trimethoprim-sulfamethoxazole (0.002-32 mg/L) for susceptibility testing on the *Listeria* isolates as per the CLSI M45 A2 document [18]. Clinical breakpoint for penicillin of ≤ 2 mg/L and for trimethoprim-sulfamethoxazole of ≤ 0.5/9.5 mg/L is interpreted as ‘sensitive’ as per this document. All three isolates had MIC value for both these antimicrobials in ‘sensitive’ range.

**Figure 3:**
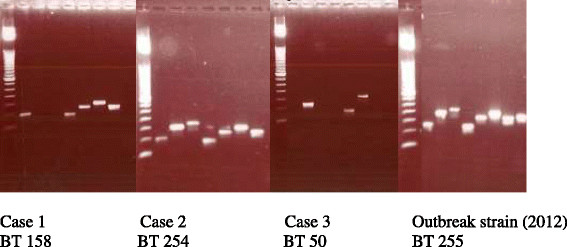
**Results of binary typing (BT) of the isolates.**

It is mandatory to report cases of listeriosis (invasive and non-invasive) in all jurisdictions in Australia [19]. Public health officers assess each of these notified cases to determine the epidemiological links and the necessity for carrying out further investigations such as food sampling, tracing the food borne source etc. Molecular analysis with binary typing and Multiple-Locus Variable number tandem repeat Analysis (*MLVA*) is performed in the state public health laboratory on all clinically significant *Listeria* isolates. Binary typing (BT) is a polymerase chain reaction (PCR) detection method of 8 gene loci on the *Listeria* genome. In binary typing, each strain is assigned a signature based on the presence (1) or absence (0) of a set of defined DNA sequences. The results of BT are combined with MLVA typing (involves testing of another 9 gene loci) to provide laboratory evidence for an outbreak. The isolates also undergo pulsed field gel electrophoresis (PFGE), multilocus sequence typing and molecular serotyping at another reference laboratory in Australia in liaison with state public health authorities. In our cases molecular analysis (Figure 3) revealed the following BT types: BT 158 (case 1), BT 254 (case 2) and BT 50 (case 3). All three cases were molecularly unrelated to the listeriosis outbreak in NSW in 2011–2012. A national network of foodborne diseases epidemiologists called OzFoodNet exists in Australia, which coordinates the analysis of the epidemiological data and molecular typing for determination of clusters and outbreaks. Public health unit case management was done on all three cases (Table 1).

**Table 1:** **Public Health unit’s case management and investigation of the three cases**

	Case 1	Case 2	Case 3
Binary type (BT)	158	254	50
MLVA type	04-17-16-05-03-12-14-00-16	03-16-21-05-03-05-15-00-18	03-12-00-00-03-00-00-00-17
Pulse Field Gel Electrophoresis	4 T: 4: 1	50A: 44A: 1	Not done
Serotype (Molecular)	1/2b, 3b, 7	4b, 4d, 4e	Not done
MLST type	3	1	299
PHU Case management	Yes	Yes	Yes
Questionnaire/Interview	Yes	Yes	Yes
Environmental evaluation	No	No	No
Potential sources identified	Uncooked sausages and meat	None	None
Case definition of Listeriosis (NSW Health)	Yes	No	No

MLVA- Multiple-Locus Variable number tandem repeat Analysis; MLST- Multi locus sequence typing, PHU- Public Health Unit.

The recommended therapeutic management of invasive listeriosis is intravenous administration of penicillin or ampicillin with or without an aminoglycoside. The added value of gentamicin is disputed and based solely on alleged in-vitro synergism [20]. The duration of therapy for focal infections is based on evidence from small case series. Optimal duration of therapy is unknown and should be based on person’s immune status and response to antimicrobials. Both patients with septic arthritis and graft infection received 6 weeks of intravenous therapy. Debridement or replacement of prosthetic material is generally considered necessary for cure, which was not performed in our case given her underlying morbidities. Thus far case 1 patient has had a good outcome. Case 2 had treatment with almost complete removal (small piece adjacent to anastomotic site left behind) of the infected graft and drainage of the collection which has been shown to have good outcomes [9],[21]. Case 3 was managed with antibiotics alone after drainage of abscess.

The finding that only Case 1 had a history of gastroenteritis highlights the fact that gastrointestinal symptoms may not be present in all patients. Asymptomatic and subclinical infections are known to occur in immunocompetent patients [22]. We speculate that case 2 and 3 may have had sporadic subclinical infections in the past with carriage of the organism in the gastrointestinal tract. Up to 5% of healthy adults can have asymptomatic carriage of *Listeria* [23]-[25]. Diagnosis of these focal infections can be extremely challenging based on their clinical presentation. However modern techniques in the laboratory like MALDI-TOF have vastly improved the detection of such infections especially when they are polymicrobial, as was demonstrated in cases 2 and 3.

It also raises the importance of providing dietary advice to patients who are undergoing chemotherapy or other immunosuppressive therapy, such as mycophenylate mofetil or TNF inhibitors. Whilst consumption of raw meat is uncommon in Australian society, large scale outbreaks of listeriosis have also occurred even with consumption of hard and soft cheeses in other countries and Australia [3],[22],[26]. We recommend extending the advice that is provided to pregnant women regarding the dangers of raw and unpasteurised foods and soft cheese to all patients undergoing immunosuppression. There is a need for heightened awareness amongst clinicians of unusual organisms causing these focal infections where requirement for obtaining a microbiological specimen is crucial for diagnosis. Treatment is often prolonged and inappropriate therapy may lead to progression of the infection.

## Conclusion

Although localised and focal infections with *Listeria* are rare, they present with challenges associated with diagnosis and may not have clear risk factors for their development. A thorough clinical history is vital and improved microbiological techniques have facilitated their detection. We suggest giving appropriate dietary advice to immunocompromised patients.

## Consent

Written informed consent was obtained from all patients for publication of this Case report and any accompanying images. A copy of the written consent is available for review by the Editor of this journal.

## Authors’ original submitted files for images

Below are the links to the authors’ original submitted files for images. Authors’ original file for figure 1 Authors’ original file for figure 2 Authors’ original file for figure 3
