# Supplementary figures and images for: Uncommon manifestations of Listeria monocytogenes infection
Source: BMC Infect Dis. 2014 Dec 3;14:641. doi: 10.1186/s12879-014-0641-x (PMC4273458; doi:10.1186/s12879-014-0641-x)

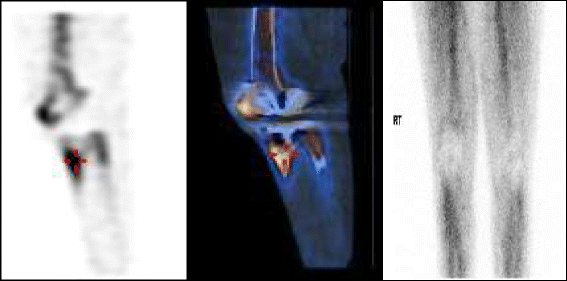

Supplement: Supplementary file 1 — Authors’ original file for figure 1 [file 12879_2014_641_MOESM1_ESM.gif]

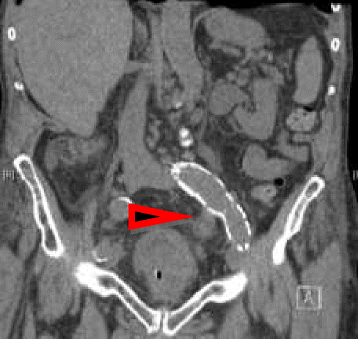

Supplement: Supplementary file 2 — Authors’ original file for figure 2 [file 12879_2014_641_MOESM2_ESM.gif]

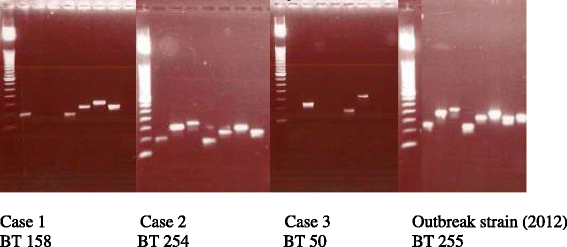

Supplement: Supplementary file 3 — Authors’ original file for figure 3 [file 12879_2014_641_MOESM3_ESM.gif]
